# Short-Wave Sensitive (“Blue”) Cone Activation Is an Aggravating Factor for Visual Snow Symptoms

**DOI:** 10.3389/fneur.2021.697923

**Published:** 2021-08-19

**Authors:** Jenny L. Hepschke, Paul R. Martin, Clare L. Fraser

**Affiliations:** Faculty of Health and Medicine, Save Sight Institute, The University of Sydney, Sydney, NSW, Australia

**Keywords:** visual snow, palinopsia, migraine, positive persistent visual disturbance, thalamocortical dysrhythmia, colour filter

## Abstract

**Background and Purpose:** Visual Snow (VS) is a disorder characterised by the subjective perception of black-and-white visual static. The aetiology of this condition is not known. In our previous work we suggested that there is a link between short-wave (S or “blue” cone) signals and severity of visual snow symptoms. Therefore we aimed to further characterise this potential link.

**Methods:** Patients (*n* = 22) with classic VS based on the diagnostic criteria and healthy controls (*n* = 12), underwent Intuitive Colorimetry (IC) testing (Cerium Visual Technologies). Twelve hue directions (expressed as angle in CIE 1976 LUV space relative to D65) were rated on a five-point scale from preferred (relieving, positive score) to non-preferred (exacerbating, negative score), and overall preferred and non-preferred angles were chosen.

**Results:** A non-preferred violet region near the tritanopic confusion line / S-cone axis (267 deg.) was strongly associated with exacerbation of VS symptoms (range 250–310 deg, mean 276 ± 16, *n* = 20, Rayleigh *p* < 0.001). Two subjects with non-preferred region > 90 deg from mean were considered as outliers. Median rank at hue angle 270 deg was significantly lower than at angle 90 (−1.5 vs. 0.0, *p* < 0.001, Wilcoxon non-parametric rank-sum test). Patients showed preference for one of two spectral regions which relieved VS symptoms: orange-yellow (range 50–110 deg., mean 79 ± 24, *n* = 14) and turquoise-blue (range (210–250 deg., mean 234 ± 27, *n* = 8).

**Conclusion:** Our results show that visual snow symptoms are exacerbated by colour modulation that selectively increased levels of S-cone excitation. Because S-cone signals travel on primordial brain pathways that regulate cortical rhythms (koniocellular pathways) we hypothesis that these pathways contribute to the pathogenesis of this disorder.

## Introduction

Visual Snow (VS) refers to the persistent visual experience of static in the whole visual field of both eyes likened to “static analogue television noise” (1) and was originally reported as a positive visual phenomena experienced by patients with migraine (2). The visual snow syndrome (VSS) is classified based on a set of diagnostic criteria which capture the spectrum of pathology of this condition (3, 4). Specifically it is defined as flickering fine achromatic dots with at least one associated visual symptom of palinopsia, photopsia, nyctalopia, and entoptic phenomena as well as non-visual symptoms such as tinnitus and migraine (3, 5).

Puledda et al. (5) provided a detailed phenotypical and epidemiological description of over one thousand patients with VS and VSS. Their study confirmed several aspects of VSS that had previously been characterised in smaller cohorts including the lack of gender prevalence, onset early in life and absence of relationship to prior psychotropic substance use (6–8). It is clear from all these studies that VS and VSS exists as a continuum and the frequency of associated non-visual symptoms often carries a higher symptom severity and burden of disease (5, 9, 10).

The pathophysiology underlying VS remains elusive, but several hypotheses exist. Cortical hyperexcitability in the visual system has been suggested as a mechanism based on detection of cortical hypermetabolism (11, 12), increased lactate presence (13) and behavioural imbalance between inhibition and excitation (9, 14). Other reports have considered mechanisms of impaired sensory processing as evidenced by hypoperfusion on SPECT (15), hyperexcitability on EEG (16), as well as evidence of reduced habituation on electrophysiological assessment (17, 18). Most recently differences in grey matter volume and resting state functional connectivity in VS patients were identified using MRI (12, 13, 19).

We have previously hypothesised that VS results from a thalamocortical dysrhythmia (TCD) of the visual system, whereby normal thalamo-cortical oscillations are disrupted by changes in the oscillatory properties of the constituent neural circuits (20). Specifically we proposed that VS is associated with abnormalities to the koniocellular (KC) pathways, which include cells that transmit short-wave (S-cone) signals serving blue-yellow colour vision. This hypothesis was based on previous observations of yellow-blue colour preferences in VS patients (8), and is broadly in line with the thalamocortical synchrony (TCS) hypothesis (21). The TCS proposes that KC activity entrains or gates cortical circuits fed by magno- and parvocellular afferent pathways, thereby rendering otherwise sub-threshold activity in these visual pathways as visual snow (22, 23).

The present study characterises the colour preferences of VS patients in more detail, with emphasis on the tritan (blue-yellow) and protan (red-green) colour axes. Our specific hypothesis is that S-cone activation, and resultant central koniocellular pathway modulation, plays a crucial role in the pathogenesis of visual snow syndrome.

## Materials and Methods

### Participants

Data were collected from 22 VS patients and 12 controls. Patients underwent a standardised series of questions about their associated visual and non-visual symptoms. The associated medical and psychiatric co-morbidities were reviewed or noted from past medical records. The VS participants were included only if they had a clinical diagnosis by a Neuro-ophthalmologist of typical VS according to the diagnostic criteria by Schankin et al. (3). Participants were excluded if they were taking psychiatric medication, reported epileptic symptoms or had a diagnosis of Hallucinogen-persistence perceptual Disorder (HPPD).

### Intuitive Colorimetry

All participants were tested by Intuitive Colorimetry (IC) as previously described by Wilkins et al. (24) (**Figure 3**). Participants were seated in front of an Intuitive Colorimeter Device (Cerium Visual Technologies) which illuminated a page of crowded text. The participants were asked to judge whether a change in the illumination colour had any effect on their visual snow symptoms (their “visual comfort”). Saturation in the 12 different hue directions (expressed as angle in CIE 1976 LUV space relative to D65) was slowly increased from a neutral setting, which was a white similar to daylight (CIE 1976 u′ = 0.21; v′ = 0.75) to one with a moderate strength of colour or saturation. The hue directions were rated on a five-point scale from preferred (relieving, positive score), neutral to non-preferred (exacerbating, negative score). For those hues that elicited clear exacerbation or relief of visual snow symptoms the saturation was optimised, usually by asking the patient to adjust the saturation using a manual dial. The preferred and least-preferred hues were then compared, typically by forced choice between two previously selected choices successively presented by the examiner until a chromaticity had been selected by the participant.

### Analysis

Statistical comparisons of area of preferred and least preferred spectral regions were made using non-parametric tests with Matlab. The research procedures complied with the Declaration of Helsinki and were approved by the Macquarie University ethics committee (HREC 5201800350). Participants gave written informed consent.

## Results

### Epidemiology

The VS cohort consisted of 9 female and 13 male patients with a mean age of 31.8 ± 11.3 years (range 22–61 y). The average VS symptom duration was 6.8 ± 2.5 years (range 2–40 years) with four patients reporting symptoms since early childhood. Associated visual and non-visual symptoms are summarised in Table 1.

**Table 1:** Frequency of visual and non-visual symptoms of visual snow participants in line with diagnostic criteria.

**Visual snow criteria**	
***Visual symptoms***	
Visual snow	100%
Palinopsias	95%
Enhanced entoptic phenomena	91%
Photophobia	64%
Nyctalopia	64%
***Non-visual symptoms***	
Tinnitus	86%
Migraine	59%
Tremor	46%

All of the VS cohort fulfilled the diagnostic criteria of classic achromatic visual snow (3). Associated visual symptoms were reported with the following frequency; 95% palinopsias, 91% entoptic phenomena and 64% photophobia and nyctalopia. Associated non-visual symptoms included 86% tinnitus, 59% migraine, and 46% tremor.

Healthy controls were eight female and four male volunteers with a mean age of 38.4 ± 16.2 years (range 22–74 y). In our control cohort none of the patients experienced VS nor any associated visual symptoms. Five (42%) of the healthy controls had migraines and none had tinnitus or tremor.

### Intuitive Colorimetry Preference

**Figure 1:**
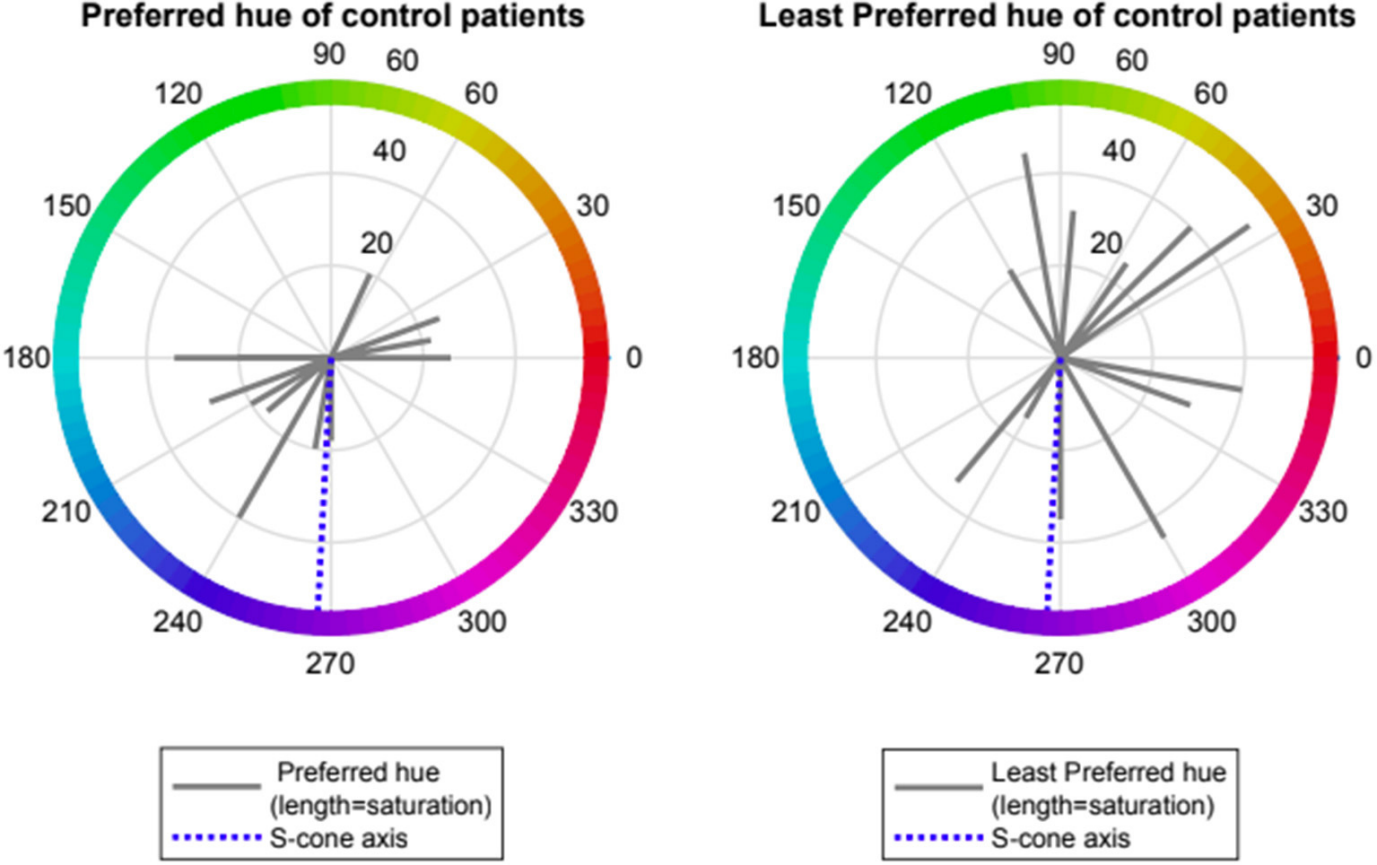
Preferred and Least preferred hue of control participants expressed as angle in CIE 1976 LUV space (red lines); dotted blue line represents the Tritanopic confusion line.

Control volunteers showed a slight preference for one of two spectral regions which provided “visual comfort” namely red-orange (range 0–70 deg., mean 12 ± 1, *n* = 4) and turquoise-blue (range (180–270 deg., mean 220 ± 35, *n* = 8). Control volunteers had no non-preferred region for “visual discomfort” (see Figure 1).

**Figure 2:**
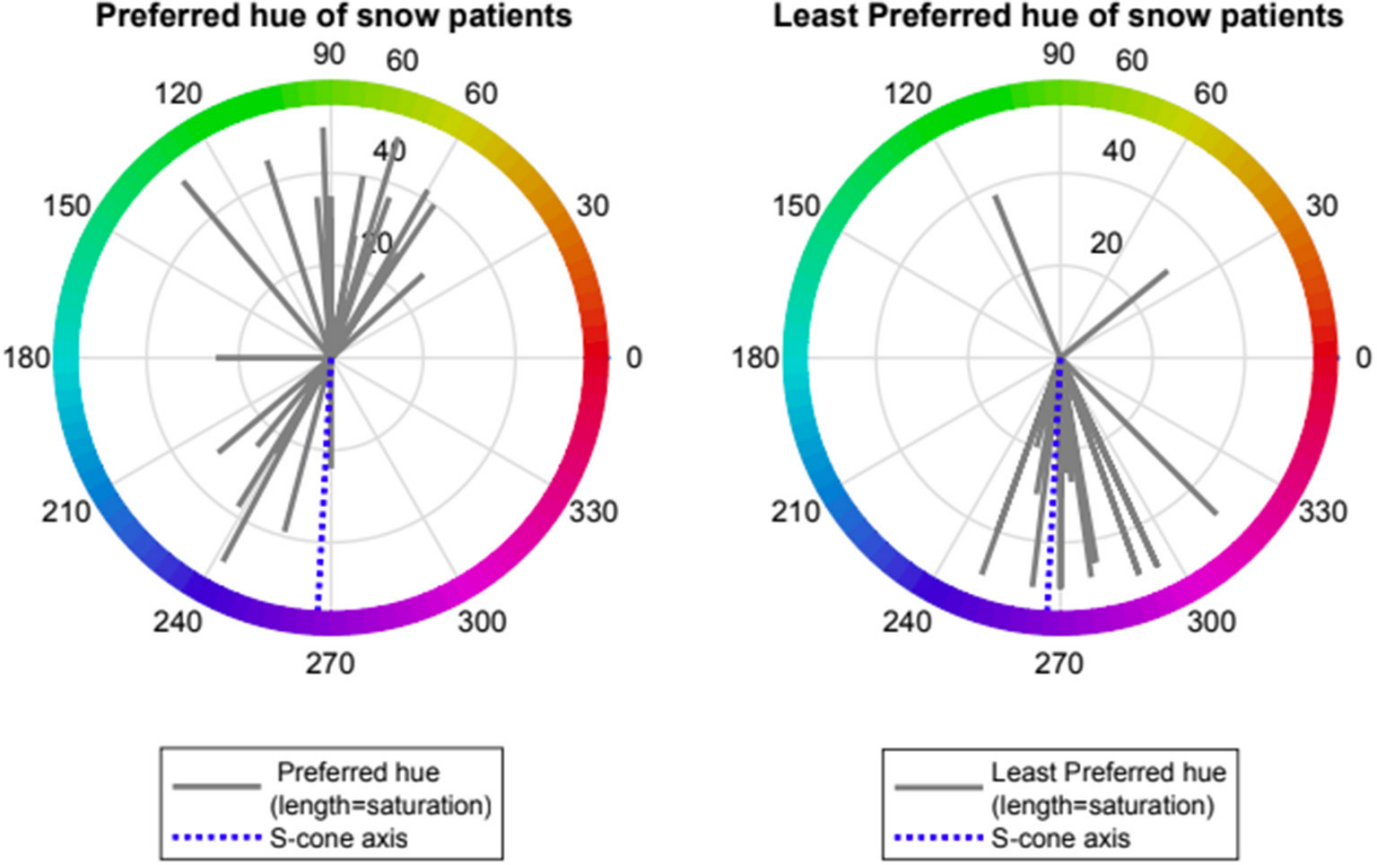
Preferred and Least preferred hue of visual snow participants expressed as angle in CIE 1976 LUV space (red lines); dotted blue line represents the Tritanopic confusion line (S-cone axis).

**Figure 3:**
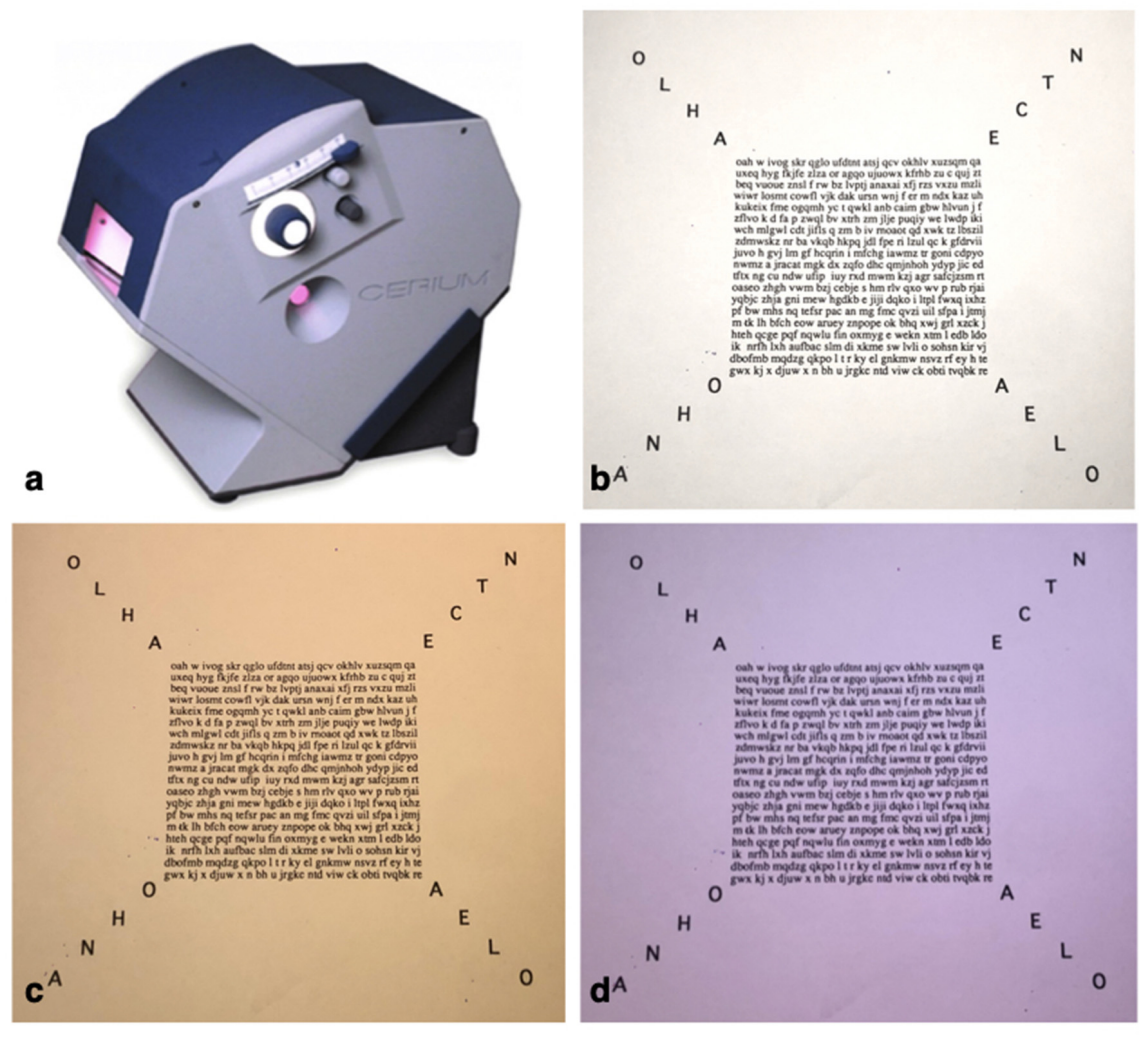
Intuitive Colorimeter **(a)**, Crowded text used in neutral/daylight setting **(b)** and the most preferred yellow hue 79 deg **(c)** as well as least preferred blue-violet hue 276 deg **(d)**.

Patients with VS showed preference for one of two spectral regions which relieved VS symptoms namely orange- yellow (range 50–110 deg., mean 79 ± 24, *n* = 14) and turquoise-blue (range (210–250 deg., mean 234 ± 27, *n* = 8). Patients with VS also showed a strong negative preference for a spectral blue-violet region which exacerbated VS symptoms (range 250–310 deg, mean 276 ± 16, *n* = 20, Rayleigh *p* < 0.001). Two subjects with non-preferred region > 90 deg from mean were considered as outliers. Median rank at hue angle 270 deg was significantly lower than at angle 90 (−1.5 vs. 0.0, *p* < 0.001, Wilcoxon non-parametric rank-sum test) (see Figures 2, 3).

## Discussion

We previously reported subjective relief of VS symptoms with yellow-blue colour filters (8). In this paper we formally classify colour preferences in VS patients compared to controls. We confirm a yellow-blue colour preferences for VS participants compared to controls, with the colour filter acting to relieve the symptoms. Most striking however was the strong negative preference or dislike for a blue-violet region (mean 276 ± 16 deg), in a direction close to the tritanopic confusion line. The tritanopic confusion line is of interest as points along this line specifically cause different levels of S-cone excitation.

The S-cones participate in the construction of a blue-yellow colour opponent channel in the retina, whereby small and large bistratified cells get ON-sign input from S-cones (via ON-type S-cone contacting bipolar cells) and OFF-sign input from medium– and long-wave sensitive cones (*via* OFF-type diffuse bipolar cells) (25). These ganglion cells project predominantly through the koniocellular (KC) layers of the lateral geniculate nucleus (LGN) to reach supragranular layers of primary visual cortex (V1). The KC pathways are part of an evolutionarily ancient group of thalamocortical pathways that include the paralemniscal somatosensory and tegmental auditory pathways, and for this reason has been characterised as a primitive visual system (21, 26–28). In contrast to KC layers, the main parvocellular (PC) and magnocellular (MC) layers of the LGN evolved relatively recently, form tightly topographically organised inputs to layer 4 in V1, and have been linked to high frequency cortical oscillations (26) and high-resolution analysis of visual inputs (21). Overall S-cones and the KC pathway are unique due to the sparse distribution of s-cones in the retina, their distinct neurotransmitter profiles and their complex and varied interconnections within the thalamus giving rise to a range of visual and non-visual pathways (25). It is important to note that S-cones also contribute to “blue-off” type responses in intrisically photosensitive melanopsin-expressing cells (29). This cell population represents a possible alternative route by which the effects we observe could be mediated.

The thalamocortical system is comprised of extensive corticothalamic connections that are arranged into networks with spatial and temporal organisation through synchronisation of oscillations thereby creating the complex pathways required for sensory perception and conscious awareness (20, 30). When the neuronal integration and synchronisation at the level of the thalamus is disrupted due to changes in specific neurons or pathways, either top-down or bottom-up, then thalamocortical dysrythmia (TCD) may arise. The model of thalamocortical dysrythmia (TCD) was first proposed by Llinás et al. (20) to explain common pathological patterns such as abnormal low-frequency theta oscillations, persistent gamma activity, and reduced resting-state alpha activity. Today, TCD is thought to contribute to diverse neuropathies depending on the localisation of the dysfunction in the thalamocortical network including migraine, neuropathic pain and tinnitus (31, 32), Parkinson's disease and depression (33, 34).

Components of the VSS have been traced to various areas in the visual system such as illusionary hallucinations can be traced to the V1 to V3 visual cortex, palinopsias can be traced to the parietal lobe coordination system and trailing as well as after-images can be located in the parietal association cortex (35). Symptoms affecting different aspect of the visual system that were traditionally held as distinct, may in fact be closely related, when considered from the perspective of TCD as a potential underlying mechanism. In addition the TCD hypothesis highlights that many non-visual symptoms affecting VS patients in other sensory domains such as migraine, tinnitus and tremor, may be explained by a single underlying pathophysiology (1, 31).

Some form of anatomical or functional disconnect between thalamus and cortex is thought to be a pre-requisite for the occurrence of TCD such as lack of afferent input in phantom pain and functional de-afferentiation in tinnitus (31, 36). Hyperexcitability of individual neurons may be a significant enough disruption to lead to TCD (37) and abnormal KC pathway input may be sufficient to drive the TCD in VSS.

In the above contexts, the clear dislike of blue light on the tritanopic confusion line we observed in the VS patients implicates S-cone activity, carried on KC pathways, enabling perception of visual snow. Our specific conjecture here is that activity in PC and MC pathways is increased by activity in KC cells, resulting in conscious awareness of sub-threshold visual stimuli. Defining a neurophysiological substrate for the pathology of VS gives further insights into this condition, helping patients and physicians work towards better treatment options. We have previously reported subjective benefit of blue-yellow coloured lenses causing improvement in VS symptoms (8). Our results have further defined the specific wavelengths implicated in VS and thus might help developing further treatment modalities that may suppress S-cone and KC activation.

## Data Availability Statement

The raw data supporting the conclusions of this article will be made available by the authors, without undue reservation.

## Ethics Statement

The studies involving human participants were reviewed and approved by Macquarie University Ethics. The patients/participants provided their written informed consent to participate in this study.

## Author Contributions

All authors listed have made a substantial, direct and intellectual contribution to the work, and approved it for publication.

## Conflict of Interest

The authors declare that the research was conducted in the absence of any commercial or financial relationships that could be construed as a potential conflict of interest.

## Publisher's Note

All claims expressed in this article are solely those of the authors and do not necessarily represent those of their affiliated organizations, or those of the publisher, the editors and the reviewers. Any product that may be evaluated in this article, or claim that may be made by its manufacturer, is not guaranteed or endorsed by the publisher.
